# Investigation on Bidirectional Pulse Electrochemical Micromachining of Micro Dimples

**DOI:** 10.3390/mi12091108

**Published:** 2021-09-15

**Authors:** Zhouzhi Gu, Xiaolei Chen, Zhongzheng Xu, Zhisen Ye, Guojun Li

**Affiliations:** 1 Jiangsu Key Laboratory of Advanced Manufacturing Technology, Huaiyin Institute of Technology, Huai’an 223000, China; guzz@nuaa.edu.cn; 2 State Key Laboratory of Precision Electronic Manufacturing Technology and Equipment, Guangdong University of Technology, Guangzhou 510006, China; xzz_gdut_edu@163.com (Z.X.); yezhisen_gdut@163.com (Z.Y.); gege_lee1997@163.com (G.L.); 3 Guangzhou Key Laboratory of Nontraditional Machining and Equipment, Guangdong University of Technology, Guangzhou 510006, China

**Keywords:** micro dimple, through-mask electrochemical micromachining, bidirectional pulse

## Abstract

Through-mask electrochemical micromachining (TMEMM) is a promising method to prepare micro dimples on the surface of metallic parts. However, the workpiece is machined one by one in traditional TMEMM. This paper introduced bidirectional pulse to TMEMM to improve the machining efficiency. Two masked workpieces were placed face to face, and connected to the ends of the bidirectional pulse power supply. Along with the change of the pulse direction, the polarities of the two workpieces were interchanged periodically, and micro dimples could be prepared on both workpieces at one time. The simulation and experiment results indicated that with bidirectional pulse mode, micro dimples with same the profile can be prepared on two workpieces at one time, and the dimension of micro dimple was smaller than that with unidirectional pulse mode. In bidirectional pulse current, the pulse frequency and pulse duty cycle played an important role on the preparation of micro dimple. With high pulse frequency and low pulse duty cycle, it is useful to reduce the undercut of micro dimple and improve the machining localization. With the pulse duty cycle of 20% and pulse frequency of 10 kHz, micro dimples with etch factor (*EF*) of 3 were well prepared on both workpieces surface.

## 1. Introduction

It has been reported that surface texture with the size of micro and nano scale plays a significant role on the regulation of surface property [1]. Micro dimples as typical kinds of surface texture are usually used to enhance the tribological properties of friction pairs and improve the cutting tool life. Wakuda et al. [2] prepared a micro-dimple array with the diameter of 100 μm, depth of 5 μm, and density of 5–20% on the friction pair’s surface, and found that the friction coefficient reduced from 0.12 to 0.10, compared to that with smooth surface. Yan et al. [3] reported that the micro dimples with the diameter from 50 to 300 µm, dimple depth from 5 to 20 µm, and area ratio from 5 to 20% could reduce the friction coefficient of sliding surfaces with oil lubrication, and the maximum reduction reached to 77.6%.

With this advantage, Hintze et al. [3] designed surface texture on the rake face of cutting tool for the machining of difficult-to-machine materials.

Several kinds of technologies have been used to generate micro dimples on metallic surface, such as micro milling, electric discharge machining (EDM), and laser machining [4,5,6]. With micro milling, the micro dimples are machined one by one, and the machining efficiency is low, meanwhile the tool could be worn. There are micro cracks and burr when micro dimples are generated with EDM and laser machining. Compared with the above methods, electrochemical machining is a promising approach to generate micro dimples with the advantages of no tool wear, no heat-affected layer, no cracks, and low cost [7,8]. Through-mask electrochemical micromachining (TMEMM) is a popular approach for the generation of micro dimples, in which the workpiece is coated of insulated photoresist with patterned through-structure as the mask, the exposed area on the workpiece will be dissolved, and then micro dimples are manufactured. With this method, micro dimples with the diameter of 30 μm were well prepared on a titanium surface [9]. It was also realized to prepare micro structure on cylindrical surface [10]. While, in traditional TMEMM process, the lithography is a necessary step, and each workpiece needs to be treated with lithography, which makes the process complicated. Researchers also proposed several methods to simplify the machining process. Zhu et al. [11] developed a modified TMEMM process, an insulated thin sheet with patterned through-structure was employed as the mask instead of the photoresist. There was no dissolution and damage on the sheet, and it could be reused during machining. Qu et al. [12] proposed a method for the fabrication of PDMS mask, and introduced the polydimethylsiloxane (PDMS) to TMEMM, realizing the preparation of micro dimples with high machining localization [13]. As the PDMS mask is flexible, it is also used to prepare micro dimples on cylindrical surface. Chen et al. [14] introduced a conductive mask instead of insulated mask to reduce the undercutting of micro dimple, and the machining localization was improved significantly. Different from the mask covered on the workpiece, Roy et al. [15] proposed to prepare a patterned mask covered on the cathode, and then placed the workpiece close proximity to the masked cathode, thus the exposed pattern on the cathode could be copied on the workpiece. During the process, the cathode was not dissolved, the mask has no damage, and it could be reused. Zhang et al. [16] introduced a sandwich-like electrochemical micromachining, the masked cathode was directed covered on the workpiece, and the machining mode was like a sandwich. With this mode, the distribution of current density on the workpiece was uniform, which improved the dimensional uniformity of micro dimples.

In the above literatures, several methods have been proposed to reuse the mask and to simplify the process, which were helpful to improve the machining efficiency. However, only one workpiece could be machined at one time with all the methods mentioned. It is necessary to explore a new method to further improve the machining efficiency. This paper proposed a novel method and introduced bidirectional pulse to TMEMM for the preparation of micro dimples. Two workpieces covered with masks are placed face to face, and connected to the ends of the bidirectional pulse power supply. During machining, along with the change of the pulse direction, the polarities of the two workpieces were interchanged periodically. Thus, micro dimples could be prepared on both of the workpieces at one time, which improves the machining efficiency. In this study, a mathematical model was built to analyze the evolution process of the micro dimple with bidirectional pulse, and the experiments were also performed to verify the proposed method.

## 2. Description of the Method and Numerical Simulation

### 2.1. Description of the Method

**Figure 1:**
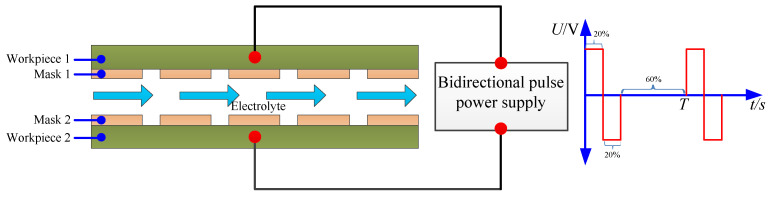
The schematic diagram of bidirectional pulse electrochemical micromachining.

Figure 1 shows a schematic diagram of the bidirectional pulse electrochemical micromachining for the fabrication of micro dimples. Two workpieces were used in the process, the patterned masks were attached on both of the workpieces, and they were placed face to face with a certain distance. During machining, the two workpieces were connected to the ends of the bidirectional pulse power supply. As the bidirectional pulse power supply was employed, the polarities of the two workpieces were interchanged from cathode to anode periodically along with the change of the pulse direction, and then the exposed area on both of the workpieces could be dissolved alternately along with the flush of electrolyte in the interelectrode gap. As the mask could be reused, and two workpieces were machined in one step, the processing cost was reduced and the machining efficiency was improved significantly, which shows a promising method in the industry.

### 2.2. Numerical Simulation

In this research, two masked workpieces were used and bidirectional pulse was introduced. A mathematical simulation model was established to analyze the difference of current density in the machining region with bidirectional pulse current and traditional unidirectional pulse current, and the sectional profile of the micro dimple was analyzed at the same time.

#### 2.2.1. Model Building

**Figure 2:**
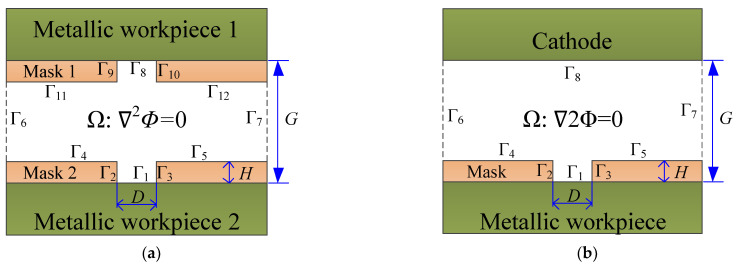
The model for the simulation. (**a**) bidirectional pulse; (**b**) unidirectional pulse.

A 2D simulation model based on the electric field mode was established, as shown in Figure 2. *H* denotes the thickness of the mask, *G* denotes the interelectrode gap, and *D* is the diameter of patterned structure in the mask. Figure 2a shows the model with bidirectional pulse current, and two masks were employed. Figure 2b shows the model with tradition unidirectional pulse current, the mask is only covered on the workpiece, and the cathode is exposed completely. During simulation, the assumption was made that: (1) the electrolyte conductivity (*σ*) is constant; (2) the electrolyte temperature (*T*) is constant; and (3) the current efficiency (*η*) is constant with 1.

According to Laplace’s equation, the electric potential *φ* is described as:

∇^2^*φ* = 0 (1)

For bidirectional pulse current, the boundaries are set by:

(2) $$\{\begin{array}{c} \varphi |\Gamma_{8}=U_{1}\;\;\;\;\;\;\;\;\;\;\;\;\;\;\;\;\;\;\;\;\;\;\;\;\left(\mathrm{Workpiece}\;1\;\mathrm{boundary}\right)\\ \varphi |\Gamma_{1}=U_{2}\;\;\;\;\;\;\;\;\;\;\;\;\;\;\;\;\;\;\;\;\;\;\;\;\left(\mathrm{Workpiece}\;2\;\mathrm{boundary}\right)\\ \frac{\partial \varphi }{\partial n}|\Gamma_{2,3,4,5,6,7,9,10,11,12}=0\;\left(\mathrm{Insulation}\;\mathrm{boundaries}\right) \end{array}$$

For traditional unidirectional pulse current, the boundaries are set as follows:

(3) $$\{\begin{array}{ll} \varphi |\Gamma_{1}=U_{1} & \left(\mathrm{Workpiece}\;\mathrm{boundary}\right)\\ \varphi |\Gamma_{8}=0 & \left(\mathrm{Cathode}\;\mathrm{boundaries}\right)\\ \frac{\partial \varphi }{\partial n}|\Gamma_{2,3,4,5,6,7}=0\; & \left(\mathrm{Insulation}\;\mathrm{boundaries}\right) \end{array}$$

**Figure 3:**
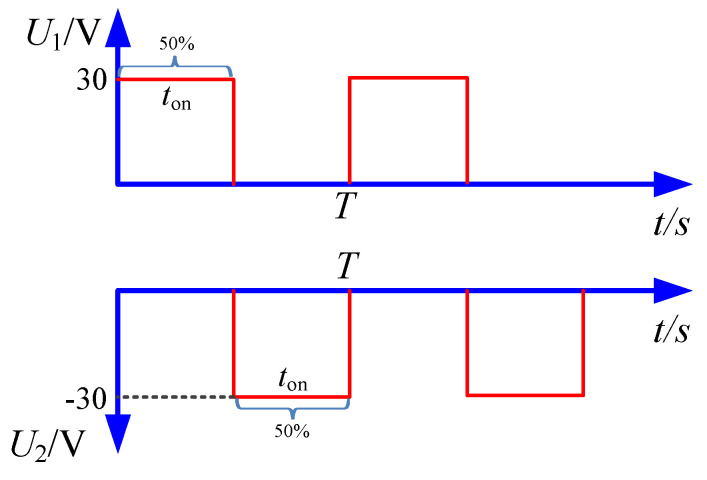
The waveform used in the simulation.

The waveform of the applied voltage used in the simulation is shown in Figure 3. In bidirectional pulse mode, *U*_1_(*t*) was applied on the workpiece 1, and *U*_2_(*t*) was applied on the workpiece 2. In unidirectional pulse mode, the *U*_1_(*t*) was just applied on the workpiece, and the cathode was zero potential.

According to the Faraday’s Law, the metal dissolution rate, *v* can be expressed as follows:

(4) $$\vec{v}=-\eta \omega \vec{i}=-\eta \omega \kappa \vec{E}$$

where *η* is the current efficiency, *ω* is the volumetric electrochemical equivalent of the metallic material, *κ* is the conductivity of electrolyte, and *E* is the electric field intensity on the workpiece.

All the models were solved by COMSOL Multiphasics software version 5.4, and the simulation parameters are listed in Table 1.

#### 2.2.2. Simulation Results

**Figure 4:**
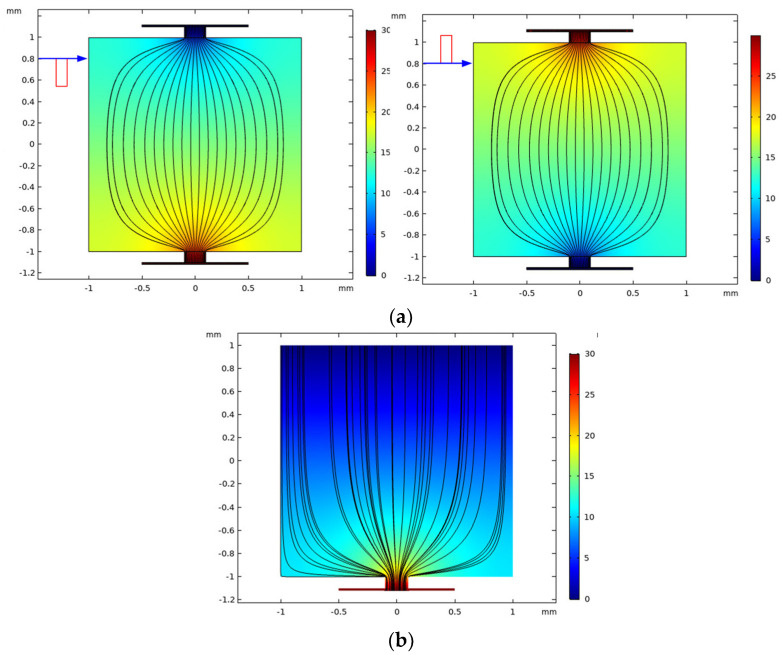
The electric line distribution with different machining modes. (**a**) bidirectional pulse; (**b**) unidirectional pulse.

Figure 4 shows the simulation result of the electric field lines distribution on the workpiece with different machining modes. It can be seen that, in the bidirectional pulse mode (Figure 4a), along with the polarity change of pulse current, the electric field lines on both workpieces are always confined by the mask through-hole. In the unidirectional pulse, the cathode is exposed, and the mask is only covered on the anode workpiece, thus the electric field lines are only confined in the mask through-hole on the anode workpiece, but they are emanative on the cathode (Figure 4b).

**Figure 5:**
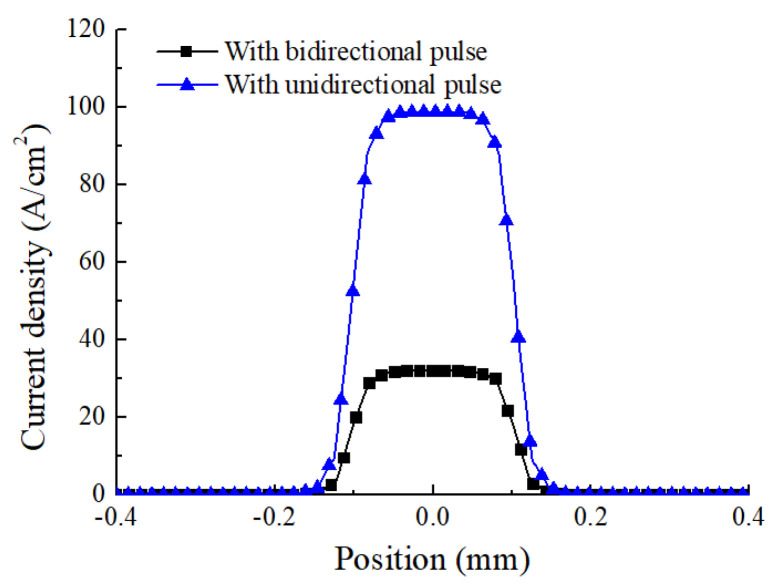
The current density distribution with different machining modes.

Figure 5 shows the simulation result of initial current density distribution on the workpiece surface with different machining modes. It can be seen that, in both machining modes, the distribution of current density is increased from the edge to the center in the mask through-hole. However, the maximum value of current density in different machining modes is different. In the bidirectional pulse mode, the maximum value of current density in the center of mask through-hole is about 25 A/cm^2^, but that with an unidirectional pulse mode is about 100 A/cm^2^. The reason for this difference could be that the masks used on both workpieces reduce the exposed area on the workpiece (Figure 4), which weaken the electric field intensity formed between the electrodes, thus the current density is reduced.

**Figure 6:**
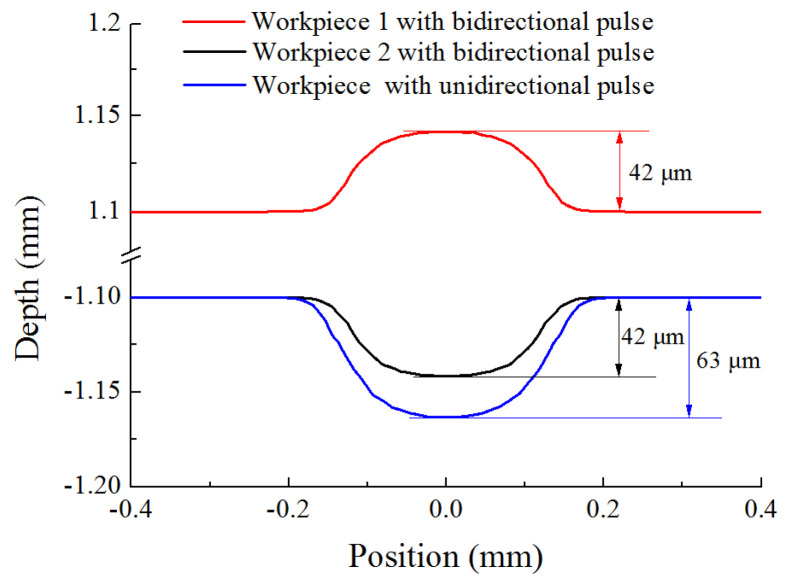
The simulation of cross-section profiles of micro dimples with different machining modes.

In order to further analyze sectional profile evolution process of micro dimple with different machining modes, simulations were conducted with the same real machining time (*t*_on_) of 8 s, and the profiles with different machining modes were compared. Figure 6 shows sectional profiles of micro dimple generated with different machining modes. It can be seen that, with the same real machining time of 8 s, a micro dimple with a diameter of 300 μm and a depth of 63 μm was generated with unidirectional pulse current. While, a micro dimple with a diameter of 250 μm and a depth of 42 μm was generated on both workpieces by using bidirectional pulse current. The dimensional difference can be explained by the difference of the current density distribution on the workpiece with different machining modes, as is analyzed in Figure 5. With the same real machining time, the current density with bidirectional pulse current was lower than that with unidirectional pulse current, leading to a low material removal amount, and the dimension of micro dimple was reduced. However, this method made it possible to machining two workpieces at one time, it reduced the preparation time for the workpiece, and the machining efficiency was improved.

## 3. Experimental

**Figure 7:**
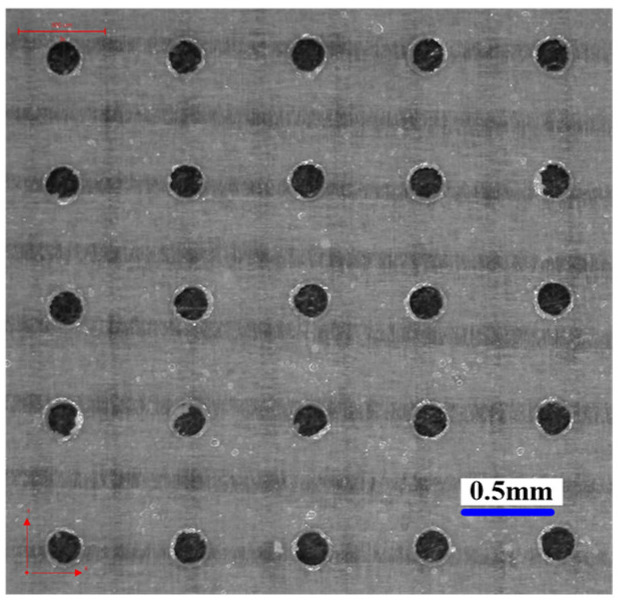
The mask used in the experiment.

Figure 7 shows the mask used in the experiment. The thickness of the mask was 100 μm, and the micro through-hole in the mask was machining with micro milling, the diameter of micro through-hole in the mask was 200 μm, and the center distance between the micro though-hole was 700 μm. A bidirectional pulse power supply (SOYI-30030DM, Shanghai SOYI Electronic Technology Co., LTD, Shanghai, China) was employed as the power supply. The 3D profiles of the micro dimples were examined by using a confocal laser-scanning microscope (CLSM, Olympus LEXT OLS4000, Japan).

In general, machining localization reflects the machining quality of a micro dimple. In this paper, etch factor (*EF*) is introduced to evaluate the machining localization of micro dimple.

(5) $$EF=2h/\left(D_{1}-D\right)$$

where *h* is the depth of micro dimple, and *D*_1_ is the diameter of micro dimple, and *D* is the diameter of micro through-hole in the mask. High *EF* means a low undercut in the diameter of micro dimple, and the machining localization is improved.

**Figure 8:**
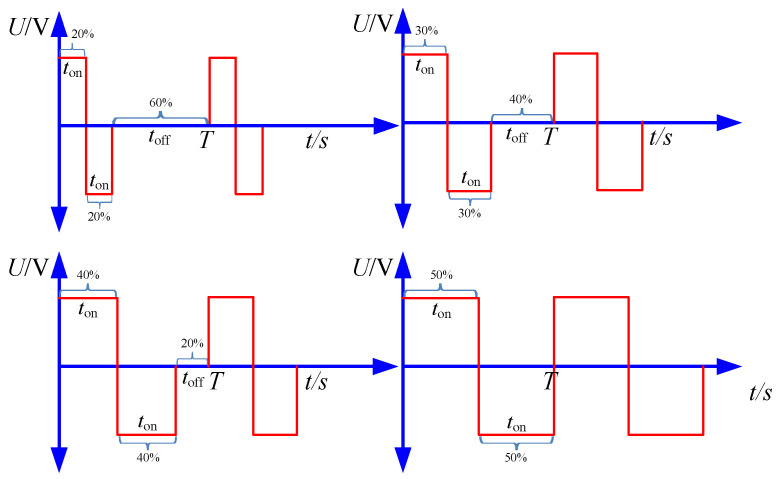
The schematic diagram of bidirectional pulse with different pulse duty cycles.

In the experiments, machining parameters are set as listed in Table 2. The pulse frequency ranged from 4 kHz to 10 kHz, and pulse duty cycle ranged from 20% to 50%. The diagram of pulse waveform is shown in Figure 8. In each pulse duty cycle, positive pulse voltage and negative pulse voltage were supplied in turn, then a pulse off-time was followed, thus the material on the two workpieces was dissolved alternately.

## 4. Results and Discussion

### 4.1. The Comparison of Micro Dimple Generated with Different Modes

In order to compare the difference of micro dimples generated with different modes, the comparison experiments were designed with the same machining parameters: the applied voltage of 30 V, pulse duty cycle of 20%, pulse frequency of 4 kHz, and the real machining time of 15 s.

**Figure 9:**
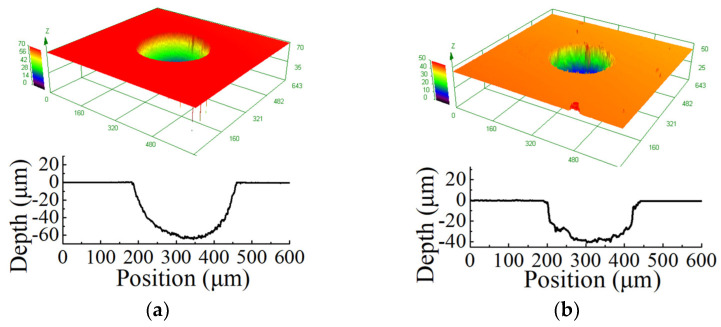
Micro dimple generated with different modes. (**a**) unidirectional pulse; (**b**) bidirectional pulse.

Figure 9a,b show 3D profiles of micro dimples generated with unidirectional pulse current and bidirectional pulse current, respectively. It can be seen that, with the same machining parameters, the diameter of micro dimple was about 275.8 μm, and the depth was about 60.2 μm by using unidirectional pulse current. When a bidirectional pulse current was used, the diameter of a micro dimple was about 250 μm, and the depth was about 40 μm. There was a dimensional difference of 20 μm between the two machining modes, and the material removal amount with was bidirectional pulse current was slightly lower than that with unidirectional pulse current. The reason was because the current density distribution on the workpiece changed when two masks were employed, as explained in the simulation. While, through the calculation with Equation (5), it can be found that both of the *EF* were about 1.6. This indicated that, although the material removal amount with bidirectional pulse current was slightly lower than that with unidirectional pulse current, the machining localization had no change. In addition, the bidirectional pulse current realized the machining of micro dimples on two workpieces at one time, the machining efficiency was improved. In order to further investigate the machining process with bidirectional pulse current, the following experiments were performed.

### 4.2. The Effect of Pulse Frequency on the Generation of Micro Dimple

In pulse current, the pulse frequency was a key parameter that affects the machining process. In order to investigate the effect of pulse frequency on the machining process with bidirectional pulse current, the pulse frequency was set with 4 kHz, 6 kHz, 8 kHz, and 10 kHz, and other machining parameters were set with an applied voltage of 30 V, a pulse duty cycle of 20%, and a real machining time of 15 s.

**Figure 10:**
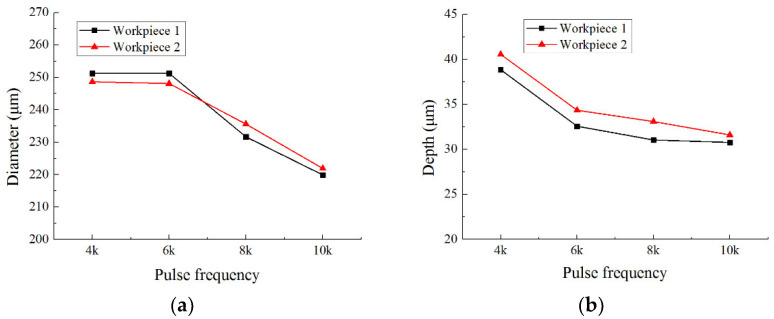
The effect of pulse frequency on the dimension of micro dimples. (**a**) diameter; (**b**) depth.

**Figure 11:**
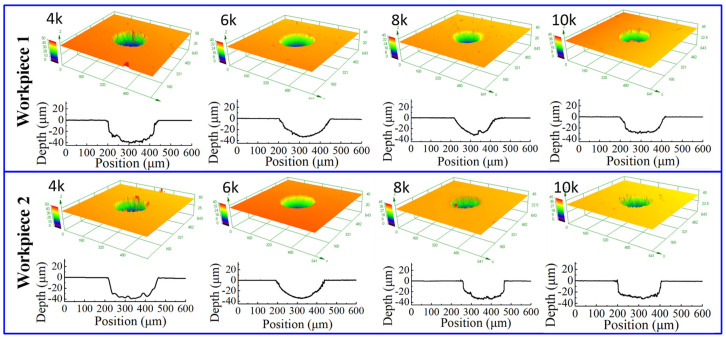
The 3D profile of micro dimple generated with different pulse frequencies.

**Figure 12:**
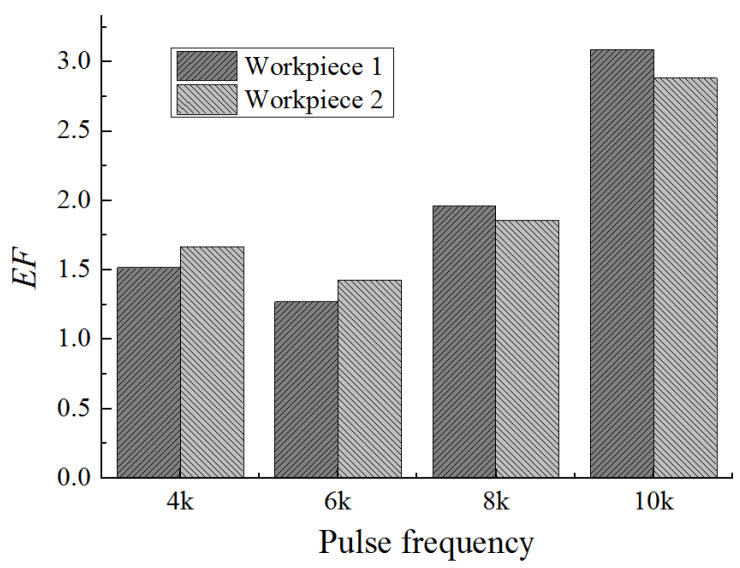
The *EF* of micro dimples generated with different pulse frequencies.

Figure 10 shows the dimensional change of micro dimples generated on the two workpieces with different pulse frequencies, and Figure 11 shows the 3D profiles of micro dimples accordingly. It can be seen that, with the same pulse frequency, the dimensional difference of micro dimples between the two workpieces was insignificant, indicating that the bidirectional pulse current could realize the machining of micro dimples on two workpieces as well as ensure the dimensional uniformity. While, with the pulse frequency increased from 4 kHz to 10 kHz, the diameter of micro dimple had significant change (Figure 10a), it decreased from 250 μm to 220 μm, and the difference value reached 30 μm. In comparison, the change of depth was not obvious (Figure 10b), it decreased from 40 μm to 32 μm, and the difference value was only 8 μm. Accordingly, Figure 12 shows the *EF* of micro dimple generated with different pulse frequencies. It can be found that the *EF* increased from 1.5 to 3 with the pulse frequency increased from 4 kHz to 10 kHz, and there was a maximum *EF* of 3 at the pulse frequency of 10 kHz. It indicated that, with the pulse frequency of 10 kHz, the undercut in diameter was weakened and the machining localization was enhanced. The reason for the effect of pulse frequency could be explained as follows.

It has been reported that the there is an obvious passive region when stainless steel 304 dissolved in NaNO_3_ solution [17], which promotes the formation of oxidation film on the workpiece surface in low current density, and hinders the material dissolution. When pulse current is used, the material dissolution includes the following processes in each pulse cycle: formation of oxidation film, break of oxidation film, dissolution of material, and product removal. The factors that affect the break of oxidation film were machining time and current density. If the on-time in one pulse cycle is short or the current density is low, the passivation process is enhanced and the break of the oxidation film will be weakened, which reduces the material dissolution. For the same pulse duty cycle of 20%, the on-time in one pulse cycle is decreased from 50 μs to 20 μs while the pulse frequency increased from 4 kHz to 10 kHz. Thus, the material dissolution amount was reduced with the increasing pulse frequency, leading to the dimensional reduction. In addition, Figure 5 in the simulation shows that the current density on the workpiece was gradually increased from the edge of mask to the center. Low current density was useful for the formation of passive region, cooperated with the high pulse frequency, the material dissolution at the edge of micro dimple was further weakened, leading to an obvious reduction of diameter at the pulse frequency of 10 kHz. While, in the center of micro dimple, the current density was high, the oxidation film could be broken relatively easy, and the change in depth with different pulse frequencies was not as significant as that in diameter.

From the experiment and analysis, it can be concluded that, with a bidirectional pulse current, micro dimples on two workpieces can be prepared at one time, the pulse frequency played an important role on the generation of micro dimples, and the pulse frequency of 10 kHz was useful to reduce the undercut of micro dimple in diameter, and improve the machining localization.

### 4.3. The Effect of Pulse Duty Cycle on the Generation of Micro Dimple

Besides pulse frequency, pulse duty cycle is another parameter that affects the machining process in bidirectional pulse current. In order to investigate the effect of pulse duty cycle on the machining process with bidirectional pulse current, the pulse duty cycle was set with 20%, 30%, 40%, and 50%, and other machining parameters were set with an applied voltage of 30 V, a pulse frequency of 10 kHz, and a real machining time of 15 s.

**Figure 13:**
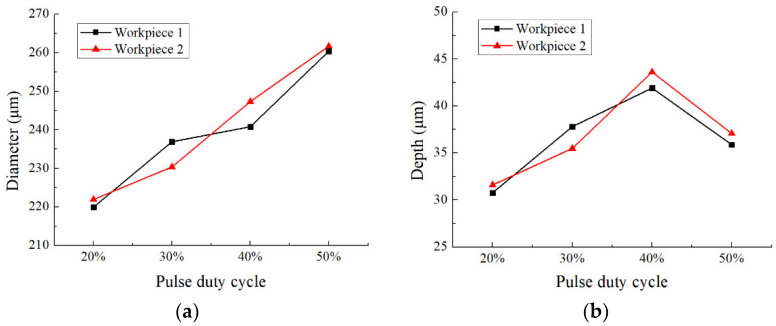
The effect of pulse duty cycle on the dimension of micro dimples. (**a**) Diameter; (**b**) Depth.

**Figure 14:**
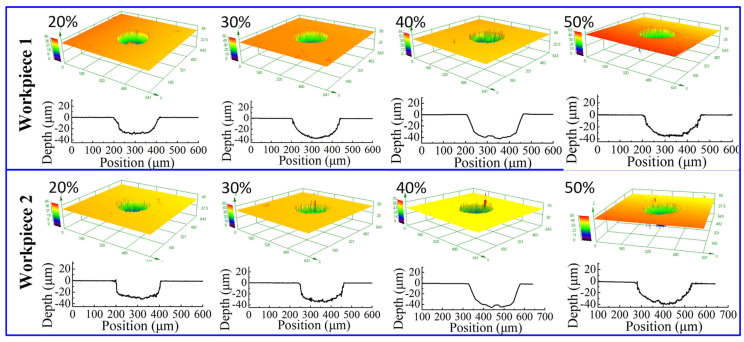
The 3D profile of micro dimple generated with different pulse duty cycles.

**Figure 15:**
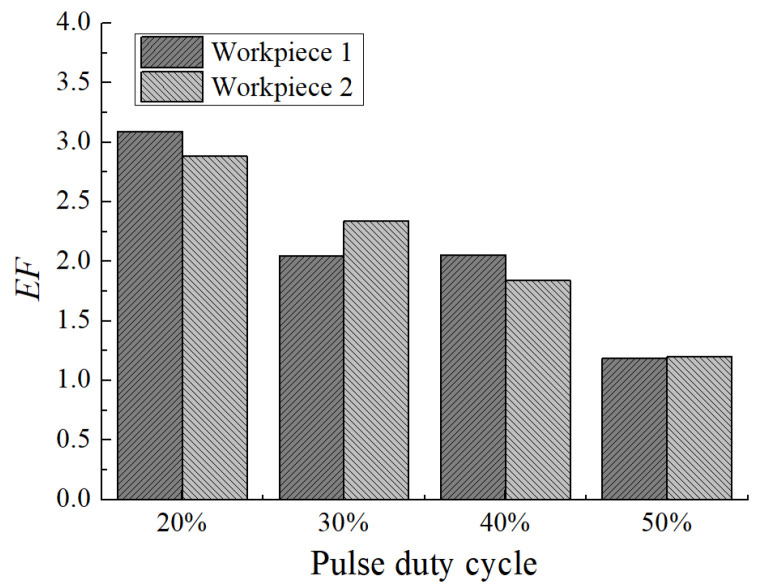
The *EF* of micro dimples generated with different pulse duty cycles.

Figure 13 shows the dimensional change of micro dimples generated with different pulse duty cycles on the two workpieces, and Figure 14 shows the 3D profiles of micro dimples accordingly. It can be seen that with the pulse duty cycle increased from 20% to 50%, the diameter of micro dimple increased from 220 μm to 260 μm, and the difference value reached to 40 μm. While, the depth of micro dimple increased from 30 μm to 42 μm when the pulse duty cycle increased from 20% to 40%, then decreased to 36 μm when the pulse duty cycle further increased to 50%, and the maximal depth difference was 12 μm. In addition, from Figure 15, it can be found that *EF* was about 3 with the pulse duty cycle of 20%, and it decreased when the pulse duty cycle increased. When the pulse duty cycle increased to 50%, the *EF* reduced to about 1.2. It shows that the machining localization was significantly weakened when the pulse duty cycle increased.

The reason for the dimensional change with different pulse duty cycles can be explained as follows.

With the pulse duty cycle increased, the on-time in one pulse cycle was increased, the break of oxidation film and the material dissolution was promoted, and both diameter and depth were enlarged, which reduced the machining localization, and the *EF* was reduced with pulse duty cycle increased. In addition, from the waveform of pulse current shown in Figure 8, it can be found that, with a bidirectional pulse current, in one pulse cycle, when one workpiece was anode (material dissolution), the other one was cathode (hydrogen generation) and the process was alternate. Then, it was followed by a pulse off-time, no electrochemical reaction took place in pulse off-time, and its benefitable for the removal of the electrolytic by-product generated on the exposed workpiece in the mask through-hole, as well as the material dissolution in the next pulse period. While, with the pulse duty cycle of 50%, it can be found that there was no pulse off-time, and material dissolution and hydrogen generation were alternate on one workpiece. Thus, the electrolytic by-product would accumulate in the mask through-hole, especially the hydrogen bubbles generated in the mask through-hole, which reduced the material dissolution in the next pulse period. That is why the depth of micro dimple was decreased with the pulse duty cycle of 50%.

**Figure 16:**
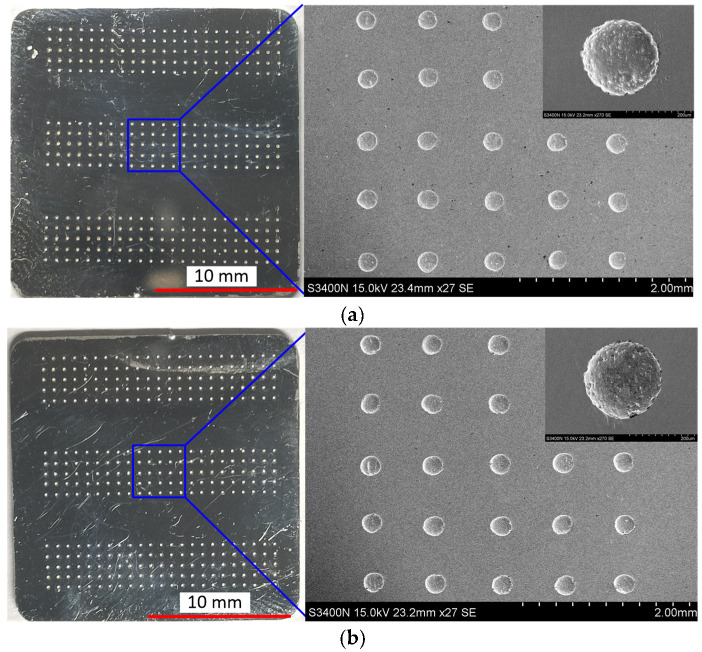
Micro dimples generated on two workpieces at one time with the optimized parameters. (**a**) Workpiece 1; (**b**) Workpiece 2.

According to the simulation and experiment research, it can be concluded that the bidirectional pulse TMEMM is a high-efficient method to prepare micro dimples, and, with the applied voltage of 30 V, pulse frequency of 10 kHz, and pulse duty cycle of 20%, micro dimples with high machining localization were well prepared in two workpieces at one time, as shown in Figure 16.

## 5. Conclusions

This paper introduced a bidirectional pulse current to TMEMM so it could prepare micro dimples on two workpieces at one time, and, based on the simulation and experiment, conclusions can be obtained as follows.

1. The simulation result indicated that, with a bidirectional pulse mode, micro dimples with the same profile can be prepared on two workpieces at one time. Additionally, the dimension of micro dimple was smaller than that with unidirectional pulse mode.
2. The experimental results agreed well with the simulation result. There was a dimensional difference of 20 μm between the two machining modes, but the machining localization has no change.
3. With a bidirectional pulse mode, the pulse frequency played an important role on the preparation of micro dimple. With the pulse frequency of 10 kHz, the undercut in diameter of micro dimple was reduced, and machining localization was significantly improved.
4. With the pulse duty cycle increased, the dimension of micro dimple was enlarged, but the machining localization was reduced. A pulse duty cycle of 20% was suitable for the fabrication of micro dimple with high machining localization.

## Figures and Tables

**Table 1:** The parameters for the simulation.

Parameter	Value
*κ*, Electrolyte conductivity	15 S/m
*U*, Applied voltage	30 V
*ω*, Volumetric electrochemical equivalent	0.035 mm^3^/(A·s)
*η*, Current efficiency	1
*D*, Diameter of the micro through-hole	200 μm
*H*, Thickness of the mask	0.1 mm
*t*_on_, real machining time	10 s
*T*, Pulse cycle	0.01 s
*G*, interelectrode gap	2 mm

**Table 2:** Machining parameters.

Parameters	Value
Electrolyte concentration	12%(*wt*.%), NaNO_3_
Electrolyte temperature	25 ℃
Electrolyte pressure	0.5 MPa
Diameter of the micro through-hole	200 μm
Center distance between micro through-holes	700 μm
Thickness of the mask	100 μm
Applied voltage	30 V
Pulse frequency	4 kHz, 6 kHz, 8 kHz, 10 kHz
Pulse duty cycle	20%, 30%, 40%, 50%
Real machining time	15 s
Material of workpiece	Stainless steel 304

## Data Availability

Not applicable.
